# 245. Comparing *Enterococcus faecalis* and *Enterococcus faecium* Bloodstream Infections’ Severity and Mortality

**DOI:** 10.1093/ofid/ofad500.318

**Published:** 2023-11-27

**Authors:** Shawn Varghese, James Sanders, Marguerite Monogue, Christina Yen

**Affiliations:** UT Southwestern Medical Center, North New Hyde Park, New York; UT Southwestern Medical Center, North New Hyde Park, New York; University of Texas Southwestern Medical Center, Dallas, TX; Department of Internal Medicine, Division of Infectious Diseases, Maine Medical Center, Dallas, TX

## Abstract

**Background:**

*Enterococcus* species commonly cause bloodstream infections (BSIs) with *E. faecium* infections considered especially worrisome due to association with vancomycin resistance via expression of the vanA gene. However, it is unclear if there is an association with *E. faecium’s* drug resistance potential and increased morbidity or mortality. Here we aimed to assess the severity of disease and mortality of patients with *E. faecalis* versus *E. faecium* BSIs, including subgroup analysis of vancomycin resistant *E. faecium* (VRE).

**Methods:**

We conducted a retrospective, observational cohort study at a large urban academic center. Adult patients admitted from January 2018 to October 2022 with either *E. faecalis* or *E. faecium* BSI were included. Variables collected, determined *a priori*, included history of previous antibiotic use, malignancy, transplant status, and bacteremia source. Outcome variables of interest were ICU stay, length of stay, and mortality.

**Results:**

There was a total of 182 total cases of *E. faecalis* and *E. faecium* BSI, 123 and 65, respectively, including 6 cases with both species (Table 1). Patients with *E. faecium* had higher median Pitt bacteremia scores (2 [IQR 0-4] versus 1 [IQR 0-3], p = 0.02) and longer median length of stay (30 [IQR 12-51] versus 15 [IQR 8-27], p < 0.001). There was statistically higher all-cause mortality at time of data collection in the *E. faecium* (60% vs 41%, p = 0.01) and VRE cohorts compared with *E. faecalis* (63% vs. 41%, p = 0.02). Kaplan-Meier curve (Figure 1) demonstrates decreased survival in the *E. faecium* cohort compared to *E. faecalis* within the first 100 days after bacteremia (HR 1.84, p = 0.01). Of note, a difference in 90-day all cause mortality was only statistically different in the entire E. faecium cohort when compared to the E. faecalis cohort. (45% vs 29%, p = 0.04), in contrast to the VRE cohort (43% vs 29%, p = 0.12).

E. faecalis vs E. faecium results

**Figure:**
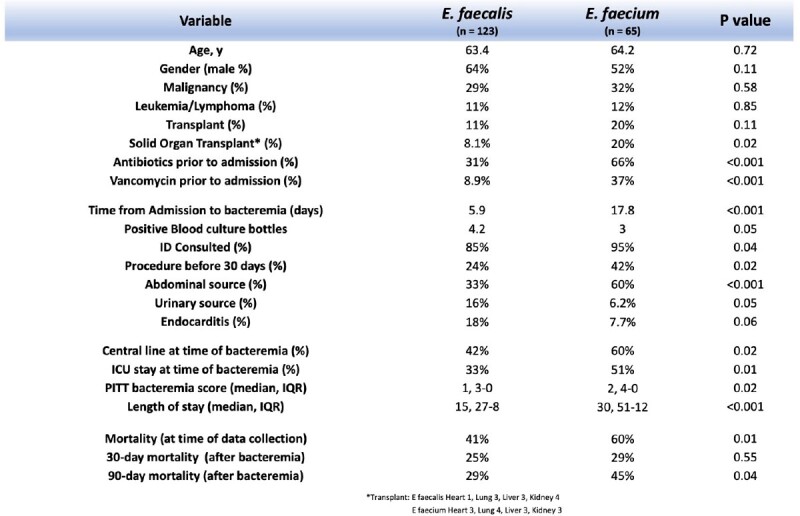

Comparison of select variables

E. faecalis vs vancomycin resistant E. faecium results

**Figure:**
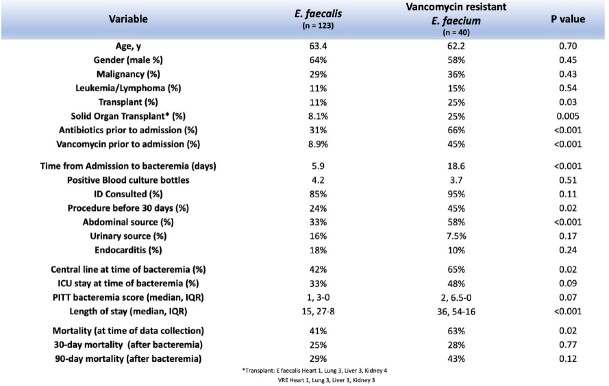

Comparison of select variables

**Figure:**
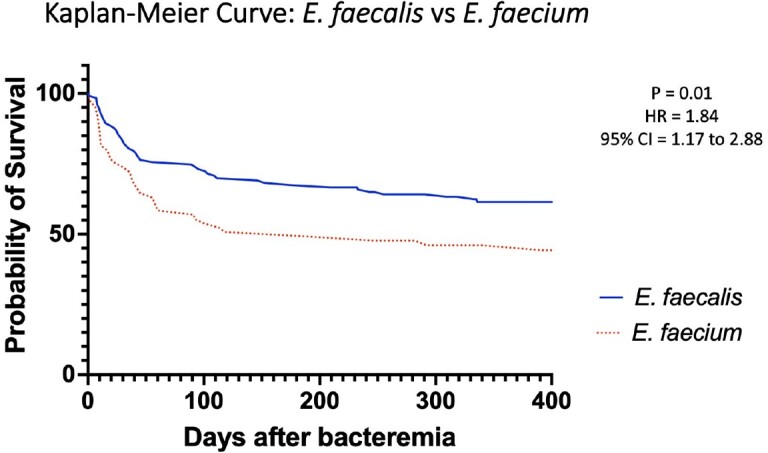

**Conclusion:**

*E. faecium* BSIs appear associated with longer, more clinically severe hospital courses and significantly increased mortality, specifically 90-day mortality when compared to *E. faecalis* BSIs. Future inquiries should assess the extent to which *E. faecium* serves as an index versus driver of poor clinical outcomes and strategies to mitigate the associated mortality.

**Disclosures:**

**James Sanders, PhD, PharmD**, Merck & Co., Inc.: Grant/Research Support|Shionogi Inc.: Grant/Research Support

